# Opioid-induced respiratory depression increases hospital costs and length of stay in patients recovering on the general care floor

**DOI:** 10.1186/s12871-021-01307-8

**Published:** 2021-03-20

**Authors:** Ashish K. Khanna, Leif Saager, Sergio D. Bergese, Carla R. Jungquist, Hiroshi Morimatsu, Shoichi Uezono, Lian Kah Ti, Roy Soto, Wei Jiang, Wolfgang Buhre

**Affiliations:** 1 Wake Forest School of Medicine, Winston-Salem, NC USA; 2 Outcomes Research Consortium, Cleveland, OH USA; 3 Universitätsmedizin Göttingen, Göttingen, Germany; 4 Stony Brook Medicine, Stony Brook, NY USA; 5 University at Buffalo School of Nursing, Buffalo, NY USA; 6 Okayama University Hospital, Okayama, Japan; 7 Jikei University School of Medicine, Tokyo, Japan; 8 National University of Singapore, Singapore, Singapore; 9 Beaumont Hospital, Royal Oak, MI USA; 10 Medtronic Inc., Mansfield, MA USA; 11 University Medical Center, Utrecht, Netherlands

**Keywords:** Respiratory depression, Healthcare utilization, Opioids, Detection, Post-operative, Patient safety, Monitoring, Costs

## Abstract

**Background:**

Opioid-induced respiratory depression is common on the general care floor. However, the clinical and economic burden of respiratory depression is not well-described. The PRediction of Opioid-induced respiratory Depression In patients monitored by capnoGraphY (PRODIGY) trial created a prediction tool to identify patients at risk of respiratory depression. The purpose of this retrospective sub-analysis was to examine healthcare utilization and hospital cost associated with respiratory depression.

**Methods:**

One thousand three hundred thirty-five patients (*N* = 769 United States patients) enrolled in the PRODIGY trial received parenteral opioids and underwent continuous capnography and pulse oximetry monitoring. Cost data was retrospectively collected for 420 United States patients. Differences in healthcare utilization and costs between patients with and without ≥1 respiratory depression episode were determined. The impact of respiratory depression on hospital cost per patient was evaluated using a propensity weighted generalized linear model.

**Results:**

Patients with ≥1 respiratory depression episode had a longer length of stay (6.4 ± 7.8 days vs 5.0 ± 4.3 days, *p* = 0.009) and higher hospital cost ($21,892 ± $11,540 vs $18,206 ± $10,864, *p* = 0.002) compared to patients without respiratory depression. Patients at high risk for respiratory depression, determined using the PRODIGY risk prediction tool, who had ≥1 respiratory depression episode had higher hospital costs compared to high risk patients without respiratory depression ($21,948 ± $9128 vs $18,474 ± $9767, *p* = 0.0495). Propensity weighted analysis identified 17% higher costs for patients with ≥1 respiratory depression episode (*p* = 0.007). Length of stay significantly increased total cost, with cost increasing exponentially for patients with ≥1 respiratory depression episode as length of stay increased.

**Conclusions:**

Respiratory depression on the general care floor is associated with a significantly longer length of stay and increased hospital costs. Early identification of patients at risk for respiratory depression, along with early proactive intervention, may reduce the incidence of respiratory depression and its associated clinical and economic burden.

**Trial registration:**

ClinicalTrials.gov, NCT02811302.

**Supplementary Information:**

The online version contains supplementary material available at 10.1186/s12871-021-01307-8.

## Background

A large majority of all adverse events in hospitalized patients transpire before arrival in the intensive care unit (ICU), including 41% of in-hospital cardiac arrest events. When these events occur, about 40% of patients die before they leave the hospital [1]. Though perceived as a low-acuity environment, the hospital general care floor is actually a common venue for critical events during a period in which patients are especially prone to developing clinical deterioration and life-threatening complications [2, 3].

An episode of respiratory depression is very common on the general care floor, occurring in up to 46% of patients [4]. Respiratory depression, if defined by hypoxemia, occurs in up to a fifth of all continuously monitored patients for at least an hour of duration of recovery after non-cardiac surgery on the general care floor [5]. These are not benign occurrences, but may be associated with a series of adverse events [4, 6–12]. Opioid-induced respiratory depression is a common variant that is associated with significant morbidity and mortality [11–14]. Urman and colleagues examined 13,389 index hospitalizations where initially opioid-free patients underwent surgery. Of the 12,218 (91%) patients who received postoperative opioids, 1111 (9.1%) were identified to have potential Opioid Related Adverse Drug Events (ORADEs), of which 52% were respiratory in nature. Furthermore, the presence of an ORADE was associated with a 55% longer postoperative length of stay, 29% lower odds of discharge home, and 2.9 times the odds of death [15]. Similarly, Kessler and colleagues showed that from an initial cohort of 36,529 patients, 98.6% received opioids, and 13.6% patients with an ORADE had a 55% longer length of stay, 36% increased risk of 30-day readmission, and 3.4 times higher risk of inpatient mortality than patients who did not experience an ORADE [14]. This extent of clinical burden is supported by other literature as well [11, 16–20]. In addition to being clinically burdensome, ORADEs are costly. Numerous studies report the additive (risk-adjusted) hospitalization cost burden of surgical patients with ORADEs to be between $4350–$8225 [14, 15, 17, 20], representing a 27–47% increase in (risk-adjusted) admission costs. Importantly, a majority of these increases in healthcare utilization and cost were assessed for all ORADEs and did not delineate differences between respiratory vs non-respiratory ORADEs. Need for postoperative oxygen as a surrogate for opioid-induced respiratory depression in the post-anesthesia care unit is also associated with significant increases in day of surgery charges, respiratory charges, total charges, hospital length of stay, reintubation, and use of invasive or non-invasive ventilatory support [21].

Recently, the international PRediction of Opioid-induced respiratory Depression In patients monitored by capnoGraphY (PRODIGY) trial identified a 46% incidence of opioid-induced respiratory depression episodes among post-surgical and medical patients receiving opioids on the general care floor [4]. A respiratory depression episode included respiratory rate ≤ 5 bpm, oxygen saturation ≤ 85%, or end-tidal carbon dioxide ≤15 or ≥ 60 mmHg for ≥3 min; apnea episode lasting > 30 s; or any respiratory opioid-related adverse event requiring intervention [4, 22]. These episodes were detected by continuous capnography and pulse oximetry monitoring using the Capnostream™ 20p or 35 portable bedside monitor (Medtronic, Boulder, CO), which collects and displays end-tidal carbon dioxide, respiratory rate, pulse oximetry, and pulse rate on a single monitor [22]. As an observational trial, the monitor alarms were silenced and the monitor screen turned off to blind healthcare providers to the monitoring data. Standard of care monitoring was performed per site protocol [4]. Compared to previous studies of respiratory depression that focused solely on post-surgical patients in one country or region, PRODIGY sought to evaluate respiratory depression among a diverse population and included both surgical and medical patients with a broad range of medical histories, and enrolled patients from 7 countries across North America, Europe, and Asia [4, 5, 23, 24].

Although the impact of general ORADEs on healthcare resource utilization and cost is well described, less is understood about the influence of respiratory ORADEs, including respiratory depression episodes, on healthcare utilization and cost. The PRODIGY trial found that across 1335 patients, adverse events requiring rescue action or prolonged hospitalization occurred more commonly in patients with ≥1 opioid-induced respiratory depression episode [4]. In addition, across all patients, mean hospital length of stay was 3 days longer [4]. Here, in an analysis of a PRODIGY sub-cohort, we performed a priori analyses to derive length of stay and cost comparisons in United States patients with and without opioid-induced respiratory depression, and evaluated factors influencing patient length of stay and hospital cost.

## Methods

### Patient population

The observational PRODIGY trial (ClinicalTrials.gov: NCT02811302, 23/06/2016) enrolled 1495 post-surgical or medical patients expected to receive parenteral opioids on the general care floor across 16 trial sites in 7 countries (United States, Japan, Singapore, Germany, France, the Netherlands, and Spain) between April 2017 and May 2018 [4, 22]. Enrolled patients included those who were ≥ 18, 20, or 21 years in United States/Europe, Japan, and Singapore, respectively, able to give informed consent, and were expected to receive parenteral opioids for post-surgical or non-surgical pain on the hospital general care floor. A full list of exclusion criteria was previously described, in which patients receiving intrathecal opioids, patients receiving end of life therapy, and post-surgical patients with an American Society of Anesthesiologist (ASA) physical status V or higher were excluded [4, 22]. Similar to previous studies, patients whose hospital stay was expected to be ≤24 h and patients who were ventilated or intubated were not eligible for enrollment [22, 23]. Patients who did not receive parenteral opioids and/or did not undergo continuous capnography and pulse oximetry monitoring were excluded from the primary study analysis, resulting in an analysis cohort of 1335 patients in 7 countries [4]. Clinical trial registration, institution approval (Institutional Review Board or Research Ethics Committee, depending on trial site) and written informed consent were completed before patients were enrolled and continuously monitored using blinded capnography and pulse oximetry monitoring (Capnostream™ 20p or 35 portable bedside monitor, Nellcor™ pulse oximetry, Medtronic, Boulder, CO) for up to 48 h. Continuous, blinded capnography and pulse oximetry monitoring began after enrolled patients received opioids on the general care floor, with standard of care spot check monitoring per study site protocol. A clinical event committee reviewed the continuous monitoring waveforms to confirm respiratory depression episodes and exclude artifacts, ultimately separating patients into groups depending on the presence or absence of ≥1 respiratory depression episode [4]. This study was approved by the Institutional Review Board or Research Ethics Committee, depending on trial site. The study protocol was performed in accordance with the Declaration of Helsinki and laws and regulations of the countries in which the clinical study was conducted, including data protection laws, the Clinical Investigation Agreement and the Clinical Investigation Plan. Institutional Review Board and Research Ethics Committees that approved this research include the following: CPP Ile de France 2 (Hopital Foch); Ethik-Kommission Medizinische Fakultät (University Hospital Bonn); Rinshoushiken Shinsa Senmon Inkai (Okayama University Hospital); The Jikei Ethics Committee (Jikei University); METC MUMC+ (University Medical Center, Maastricht); National Healthcare Group (NHG) Domain Specific Review Board (DSRB) (National University of Singapore); Comité de Ética del Hospital Clinico Universitario de Valencia (Hospital Clinico Universitario de Valencia); Western Institutional Review Board (Beaumont Hospital, Emory University, Ohio State University Wexner Medical Center, and Providence Regional Medical Center); Partners Human Research Committee (Brigham and Women’s Hospital); Cleveland Clinic Institutional Review Board (Cleveland Clinic); The MetroHealth System Institutional Review Board (MetroHealth Medical Center); University at Buffalo Institutional Review Board (University at Buffalo); and Colorado Multiple Institutional Review Board (University at Colorado).

Of the 1495 prospectively enrolled PRODIGY patients, 1335 patients underwent continuous capnography and pulse oximetry monitoring and received opioids on the general care floor, including 769 patients in the United States. This sub-analysis of healthcare utilization data, which was prospectively collected during the trial, was performed using the 769 United States patients (*N* = 566 patients outside of the United States were excluded). Within the United States patient cohort (*N* = 769), retrospectively collected cost data was unavailable for 349 patients, resulting in a final patient cohort of 420 United States PRODIGY patients for analysis of cost differences between patients with and without ≥1 respiratory depression episode. Although provision of cost data was not a requirement for site participation in the trial, the cost data for the 420 patients was collected from five United States PRODIGY trial sites (Beaumont Hospital, Royal Oak, MI; Buffalo General Medical Center, Buffalo, NY; Emory University, Atlanta, GA; MetroHealth Medical Center, Cleveland OH; The Ohio State University Medical Center, Columbus, OH). Due to confounding factors, such as differences in healthcare policies that affect patient length of stay and readmission procedures between countries, differences in healthcare cost and reimbursement systems between countries, and limited sample sizes when considering PRODIGY results on a country-specific level (*N* = 28 to *N* = 213), we chose to focus this cost analysis solely on United States PRODIGY patients, who represent the largest cohort within the hospital cost dataset (*N* = 420). Therefore, our healthcare utilization analysis included 769 United States patients, and our cost analysis included 420 United States patients.

A respiratory depression episode was defined as any of the following: respiratory rate ≤ 5 bpm, oxygen saturation ≤ 85%, or end-tidal carbon dioxide ≤15 or ≥ 60 mmHg for ≥3 min; apnea episode lasting > 30 s; or any respiratory opioid-related adverse event requiring intervention, including but not limited to: narcotic overdose, partial airway obstruction, respiratory insufficiency requiring non-invasive positive pressure, respiratory failure, upper airway obstruction, cardiopulmonary arrest, and death due to respiratory or pulmonary related complications [4, 22]. Patients’ PRODIGY score was retrospectively determined using the PRODIGY risk prediction tool, described by Khanna et al. [4]. Briefly, patients were classified as low, intermediate, or high risk for respiratory depression using the risk prediction tool, which has an AUC of 0.74 [4].

### Objectives

An a priori secondary objective of the PRODIGY trial was to compare patients with and without respiratory depression for healthcare utilization, including the following endpoints: hospital length of stay, readmission rates, post-discharge healthcare utilization, and healthcare costs [22]. Post-discharge healthcare utilization included clinic visits, urgent care, emergency department visits, and inpatient hospitalization. Healthcare utilization data was collected for patients during a 30-day follow-up call, as designed in the trial protocol [22] and as is often conducted in respiratory- and ORADE-focused studies [14, 17, 19, 20]. The 30-day window for follow-up is a widely accepted timeframe for readmissions. For example, the Centers for Medicare and Medicaid (CMS) tracks complications within the 30 day window for its Hospital Readmission Reduction Program [25]. Due to variations in healthcare practices, policies, reimbursement systems, and costs between countries, these objectives were analyzed for the largest sub-cohort in PRODIGY, patients enrolled at United States trial sites.

### Statistical analysis

Data analysis was performed using SAS v9.4 (SAS Institute Inc., Cary, North Carolina). Healthcare utilization and costs were evaluated using descriptive statistics for categorical variables (percentages and counts) and continuous variables (mean and standard deviation). Total hospital costs, reflecting the sum of fixed and variable costs incurred by the hospital, were extracted from the billing department and reported directly by United States trial sites, on a per-patient level. One trial site provided total hospital charges per enrolled patient, which we converted to cost using the current cost to charge ratio (CCR), as in the literature [26, 27]. The CCR of the facility was obtained from the Medicare hospital cost report. We multiplied the hospital charges by the CCR for the estimation of hospital cost. Hypothesis test of association was conducted using Wilcoxon rank-sum test for continuous variables. Depending on the sample size, Chi-square or Fishers exact test was used for categorical variables. Statistical significance was set at 0.05 for the two-sided *p* value.

Due to the retrospective nature of this analysis, no a priori power calculations were performed. To determine the impact of individual patients’ influence on average healthcare utilization and cost measures, outliers were identified using Cook’s Distance with a cutoff > 4/(n-k-1), where n is the number of observations and k is the number of explanatory variables [28]. Length of stay and cost were evaluated with and without these patient outliers to determine whether a subset of patients strongly influenced observed trends in length of stay and costs in PRODIGY.

### Inverse probability of treatment weighting cost analysis

Inverse probability of treatment weighting using the propensity score was generated to normalize demographic and clinical characteristics (age, sex, body mass index (BMI), race/ethnicity, smoking status, neck circumference, ASA physical status, length of surgery, opioid use, and complete history of medical conditions and diseases) between patients with and without ≥1 respiratory depression episode [29]. An inverse probability of treatment weighting generalized linear model with log link function and gamma distribution was used to examine the impact of respiratory depression episode occurrence on healthcare cost. To test the effect between respiratory depression and length of stay, an interaction term of length of stay and respiratory depression was included in the generalized linear model of healthcare cost, alongside other patient demographic and clinical factors.

### Multiple regression analysis of length of stay

To identify factors associated with patient length of stay, a multiple regression model was developed for patients with and without ≥1 respiratory depression episode in the United States. The model was developed using stepwise selection with length of stay as the dependent variable and respiratory depression, baseline patient demographics, and clinical characteristics as independent variables. A generalized linear model with log link and Poisson distribution was used for the estimates.

### Missing data

Patients with missing healthcare utilization data (*n* = 1) or with missing medical history data that prevented risk stratification by the PRODIGY score (*n* = 10) were excluded from the analysis.

## Results

### Trial cohort

**Fig. 1:**
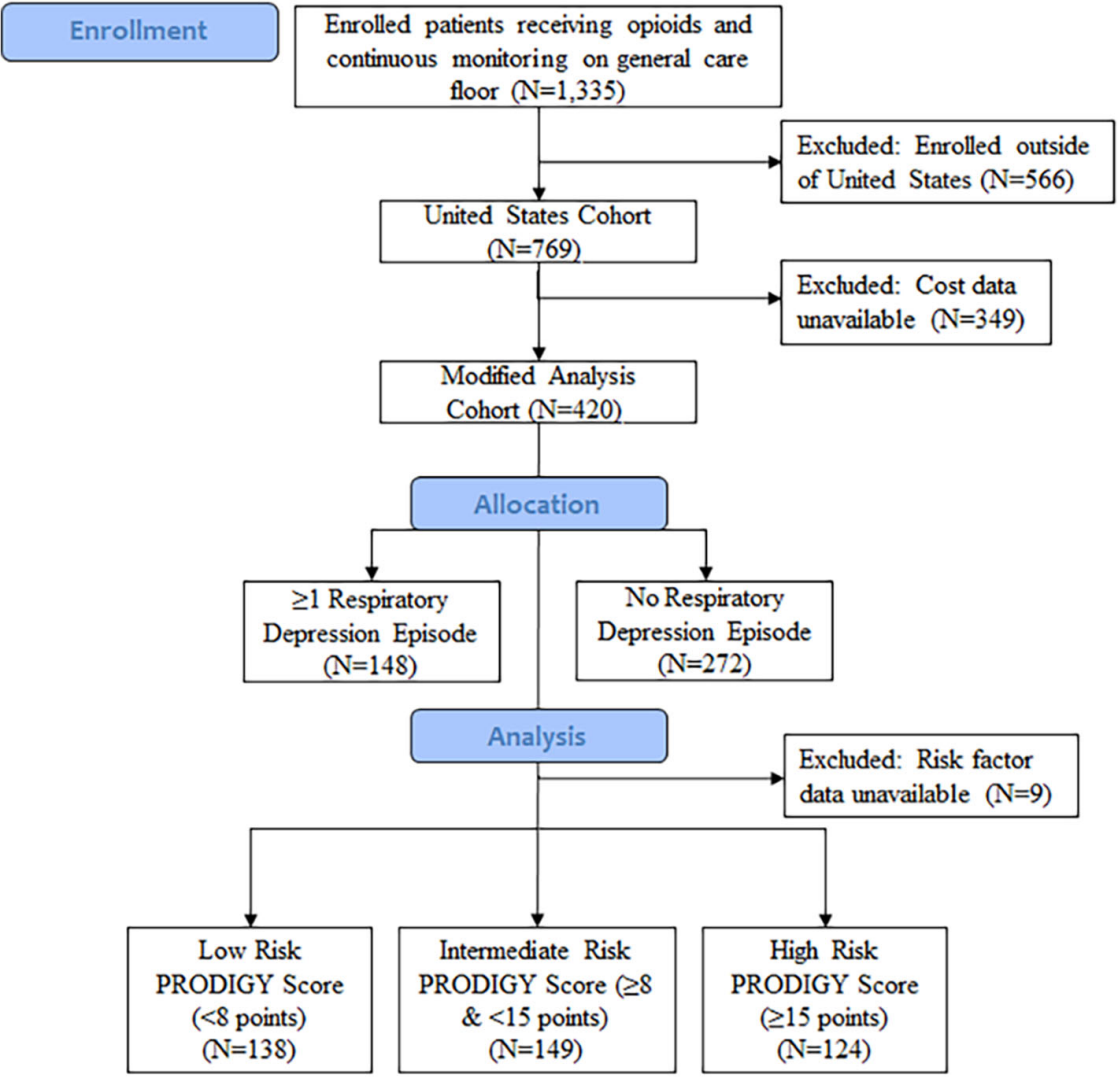
Flow chart of PRODIGY trial patients included in healthcare utilization and cost analysis

Of the 1335 patients enrolled in the PRODIGY trial who started continuous monitoring and received opioid therapy on the general care floor, healthcare utilization data was collected and analyzed for 769 patients in the United States (Fig. 1). The demographic and clinical characteristics of this cohort were described previously [4]. Thirty-seven percent (*N* = 288/769) of the patients in the United States experienced ≥1 opioid-induced respiratory depression episode during continuous monitoring. After retrospectively assigning patients’ risk for respiratory depression using the PRODIGY score (S1 Table) [4], 259, 271, and 229 patients were classified as low, intermediate, and high risk for respiratory depression, respectively. Cost data was retrospectively collected and analyzed for 420 patients enrolled in the United States, including 138, 149, and 124 patients with low, intermediate, and high risk for respiratory depression (Fig. 1).

### Post-discharge healthcare utilization

 Overall, 13% of 769 United States patients with healthcare utilization data available (*N* = 100) reported post-discharge healthcare utilization within 30 days after hospital discharge (Table 1). The majority of this post-discharge healthcare utilization involved emergency department visits or inpatient hospitalization. Although post-discharge healthcare utilization was more common in patients with ≥1 respiratory depression episode than in patients without respiratory depression episodes, this difference was not statistically significant. During the initial admission, a total of two patients (one with ≥1 respiratory depression episode) required intubation, three patients (one with ≥1 respiratory depression episode) experienced rapid response team activation, and three patients (two with ≥1 respiratory depression episode) were transferred from the general care floor to the ICU. No code blue events occurred during the trial. While patients with respiratory depression had higher frequencies of hospital readmission 7-, 15-, and 30-days after discharge, the differences between patients with and without ≥1 respiratory depression episode were not significant (Table 2).

**Table 1:** Post-discharge healthcare utilization across 769 patients enrolled in the United States with and without ≥ 1 respiratory depression episodes (%, n). Thirteen percent of United States patients with healthcare utilization data available (*N* = 100) used healthcare during the 30-days post-discharge

Healthcare Utilization	≥1 Respiratory Depression Episode	No Respiratory Depression Episodes
Any Healthcare Utilization	14.6% (42)	12.1% (58)
95% CI	10.5–18.7%	9.2–15%
Clinic visit^a^	0.7% (2)	2.1% (10)
Urgent care	0.7% (2)	0.4% (2)
Emergency department visit	7.6% (22)	6.7% (32)
Inpatient hospitalization	5.9% (17)	3.5% (17)
Other^b^	0.3% (1)	0.2% (1)
No Healthcare Utilization	85.4% (246)	87.9% (423)

Abbreviation: *95% CI* 95% confidence interval

^a^Clinic visit includes primary care, rehabilitation services, and therapy-related care

^b^Other includes telephone visit (*n* = 1) and outpatient surgery without overnight stay (*n* = 1)

**Table 2:** Healthcare utilization and cost of healthcare in United States patients with and without ≥ 1 respiratory depression episode. Outliers were identified using Cook’s Distance, resulting in exclusion of 10 patients with ≥1 respiratory depression episode and 13 patients without a respiratory depression episode from the cohort of 769 United States patients with healthcare utilization data. Within the sub-cohort of 420 patients with cost data, 5 patients with ≥1 respiratory depression episode and 6 patients without a respiratory depression episode were identified as outliers and excluded

**Healthcare Utilization**	**All Patients (*N*** **= 768)**^**a**^			**Patient Cohort Excluding Outliers (*N*** **= 745)**		
	**Patients with ≥ 1 Respiratory Depression Episode**	**Patients without Respiratory Depression Episode**	***p*-value**	**Patients with ≥ 1 Respiratory Depression Episode**	**Patients without Respiratory Depression Episode**	***p*-value**
**Length of Stay, All Patients** (Average ± SD (N))	7.1 ± 9.6 (287)	5.7 ± 6.5 (481)	.032	6.4 ± 7.8 (277)	5.0 ± 4.3 (468)	.009
**Length of Stay, PRODIGY Risk Score** (Average ± SD (N))						
Low	6.8 ± 9.4 (53)	5.2 ± 6.4 (206)	.266	5.6 ± 3.8 (52)	4.6 ± 4.0 (201)	.126
Intermediate	6.8 ± 10.7 (92)	6 ± 6.1 (178)	.497	6.5 ± 10.6 (90)	5.5 ± 4.8 (173)	.365
High	7.5 ± 9.1 (137)	6.4 ± 7.8 (92)	.322	6.7 ± 6.8 (130)	5.3 ± 3.8 (89)	.053
**7-day readmission (N,%)**	7, 2.4%	11, 2.3%	1.000	7, 2.5%	11, 2.4%	.879
**15-day readmission (N,%)**	12, 4.2%	13, 2.7%	.297	12, 4.3%	13, 2.8%	.255
**30-day readmission (N,%)**	16, 5.6%	17, 3.5%	.200	16, 5.8%	17, 3.6%	.169
**Healthcare Costs**	**All Patients (*N* = 420)**			**Patient Cohort Excluding Outliers (*N* = 409)**		
**Total Cost (USD), All Patients** (Average ± SD (N))	$23,619 ± $16,868 (148)	$19,193 ± $13,517 (272)	.006	$21,892 ± $11,540 (143)	$18,206 ± $10,864 (266)	.002
**Total Cost (USD), PRODIGY Risk Score** (Average ± SD (N))						
Low	$22,316 ± $13,679 (27)	$18,633 ± $14,050 (111)	.222	$22,316 ± $13,679 (27)	$17,705 ± $11,818 (109)	.081
Intermediate	$22,272 ± $14,661 (42)	$20,331 ± $14,594 (107)	.447	$21,665 ± $14,300 (41)	$18,858 ± $10,423 (104)	.258
High	$25,057 ± $19,490 (74)	$18,608 ± $9714 (50)	.017	$21,948 ± $9128 (70)	$18,474 ± $9767 (49)	.0495
**Propensity Weighted Cost Analysis**	**All Patients (*N*** **= 420)**			**Patient Cohort Excluding Outliers (*N*** **= 409)**		
**Overall Cost** (Average ± SD)	$23,294 ± $15,088	$20,057 ± $13,555	.013	$22,171 ± $12,727	$18,971 ± $10,725	.007
**Exponentiated Estimates** **from Generalized Linear Model** (95% CI)	1.16 (1.03–1.31)			1.17 (1.04–1.31)		

Abbreviations: *95% CI* 95% confidence interval, *N* number of patients, *PRODIGY* PRediction of Opioid-induced respiratory Depression In patients monitored by capnoGraphY, *SD* standard deviation, *USD* United States Dollars

^a^Within the United States cohort (*N* = 769), 1 patient was excluded from length of stay analysis due to missing data

### Hospital length of stay

In the United States, the average length of stay for patients with ≥1 respiratory depression episode was significantly higher compared to patients without respiratory depression episodes (7.1 ± 9.6 vs 5.7 ± 6.5 days, *p* = 0.032) (Table 2). Average length of stay was also significantly different between patients with and without ≥1 respiratory depression episode when outliers identified by Cook’s Distance were excluded from the analysis (6.4 ± 7.8 vs 5.0 ± 4.3 days, *p* = 0.009, respectively) (Table 2).

### Hospital costs

The average total hospital cost for patients in the United States who experienced ≥1 respiratory depression episode was $4426 higher (($23,619 ± $16,868 vs $19,193 ± $13,517, *p* = 0.006), compared to patients who did not experience a respiratory depression episode (Table 2). Excluding outliers, the average total hospital cost was $3686 higher for patients with ≥1 respiratory depression episode ($21,892 ± $11,540 vs $18,206 ± $10,864 for patients without respiratory depression, *p* = 0.002). For patients at high risk for respiratory depression (i.e. those with high PRODIGY score) who experienced ≥1 respiratory depression episode, the average total cost was $6648 higher ($25,057 ± $19,490 vs $18,608 ± $9714, *p* = 0.017) than high risk patients who did not experience a respiratory depression episode. Analysis excluding patient outliers also identified a significant difference between high risk patients with and without ≥1 respiratory depression episode ($21,948 ± $9128 vs $18,474 ± $9767, *p* = 0.0495), respectively (Table 1).

Propensity weighted analysis of United States patients identified a $3237 (16%) higher healthcare cost for patients with ≥1 respiratory depression episode ($23,294 ± $15,088 vs $20,057 ± $13,555 for patients without respiratory depression, *p* = 0.013), respectively (Table 2, S2 Table). Comparable results were observed upon exclusion of patient outliers, where patients with ≥1 respiratory depression episode had healthcare costs $3200 (17%) higher than patients without respiratory depression ($22,171 ± $12,727 vs $18,971 ± $10,725, respectively, *p* = 0.007) (S3 Table).

### Significant contributors to hospital costs

**Fig. 2:**
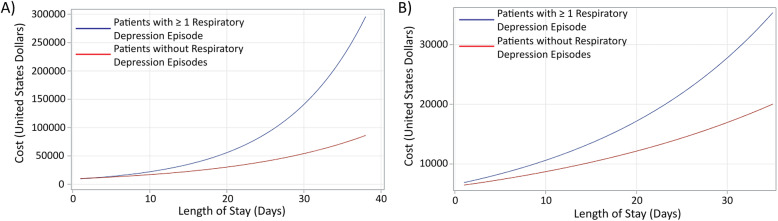
Effect of length of stay and occurrence of ≥ 1 respiratory depression episode on overall cost. **a** Overall cost, including all enrolled patients and **b** Overall cost, excluding outliers identified by Cook’s Distance. USD = United States Dollars

A generalized linear model of healthcare costs in patients in the United States, excluding outliers, with and without ≥1 respiratory depression episode, identified several variables that significantly increased healthcare costs, including length of stay (1.03, 95% CI 1.02–1.05; *p* < 0.0001), longer length of surgery (1.34, 95% CI 1.24–1.46 for surgery ≥2 - < 4 h and 1.89, 95% CI 1.69–2.12 for surgery ≥4 h, vs reference group, surgery < 2 h; *p* < 0.0001), and procedure type (nervous system 1.62, 95% CI 1.26–2.09, vs reference group, therapeutic procedures and supportive care; *p* < 0.0001) (Table 3, S4 Table). Compared to a normal BMI (20 - < 25), BMI < 20 was associated with reduced healthcare costs (0.77, 95% CI 0.58–1.02; *p* = 0.001). Similar results were observed in a generalized linear model for all patients, including outliers, where length of stay (1.06, 95% CI 1.05–1.07; *p* < 0.0001), length of stay and occurrence of ≥1 respiratory depression episode (1.04, 95% CI 1.01–1.06; *p* = 0.002), longer length of surgery (1.28, 95% CI 1.17–1.41 and 1.71, 95% CI 1.51–1.93 for surgery ≥2 - < 4 or ≥ 4 h, respectively; *p* < 0.0001), and procedure type (bone and joint 1.42, 95% CI 1.06–1.92, nervous system 1.81, 95% CI 1.35–2.41, and renal and urinary tract 2.07, 95% CI 0.93–4.58; *p* < 0.0001) significantly increased healthcare costs (S5 Table). In both analyses, the observed interaction between length of stay and occurrence of ≥1 respiratory depression episode indicates that in patients with ≥1 respiratory depression episode, as length of stay increases, healthcare costs increase exponentially, whereas in patients without respiratory depression episodes, length of stay increases healthcare costs linearly (Fig. 2a-b).

**Table 3:** Generalized linear model of healthcare costs in United States patients (excluding outliers)

Clinical Characteristic	Exponentiated estimate	95% CI	***p*** value
**Intercept**	5908.22	2704.85 – 12,905.39	<.0001
**Length of stay**	1.03	1.02–1.05	<.0001
**Respiratory depression**	1.05	0.91–1.21	.505
Length of stay* Respiratory depression	1.01	0.99–1.04	.239
**Open Surgery (vs laparoscopic)**	0.88	0.77–1.01	.067
**Length of surgery (hr)**			<.0001
≥ 2 - < 4 vs. < 2	1.34	1.24–1.46	
> 4 vs. < 2	1.89	1.69–2.12	
**BMI**			.0011
< 20	0.77	0.58–1.02	
≥ 20 - < 25	–	–	–
≥ 25 - < 30	0.90	0.80–1.00	
≥ 30 - < 35	1.01	0.90–1.14	
≥ 35	1.12	0.98–1.27	
**Procedure**			<.0001
Bone and joint	1.22	0.94–1.59	
Gastrointestinal	1.01	0.78–1.31	
Hepatobiliary	1.30	0.94–1.80	
Nervous system, skull and spine	1.62	1.26–2.09	
Obstetric and gynecological	1.06	0.80–1.41	
Renal and urinary tract	1.93	0.97–3.87	
Respiratory tract	0.98	0.47–2.02	
Therapeutic procedures and supportive care	–	–	–
Other	1.36	0.89–2.09	
Medical^a^	0.42	0.33–0.54	<.001

Scaled Deviance/Degree of Freedom (DF): 1.16

Scaled Pearson/DF: 1.25

Abbreviations: *95% CI* 95% confidence interval, *BMI* body mass index

^a^Effect of medical procedure was estimated in a separate model due to the multicollinearity between length of surgery and medical patients

### Significant contributors to hospital length of stay

A multiple regression model of patients enrolled in the United States, excluding outliers, identified multiple significant contributors to increased hospital length of stay, including use of > 1- < 4 or ≥ 4 opioids (*p* < 0.0001), surgery ≥2- < 4 h or ≥ 4 h (*p* < 0.001), high risk surgery (defined using the revised European Society of Cardiology/European Society of Anaesthesiology guidelines on non-cardiac surgery) or open surgery (*p* = 0.0005 and *p* = 0.003, respectively), respiratory depression (*p* = 0.024), hypertension (*p* = 0.011), chronic heart failure (*p* = 0.008), and sepsis (*p* < 0.0001) (Table 4). After adjusting for patient baseline characteristics, the regression model found that patients with ≥1 respiratory depression episode had a hospital length of stay 9% (95% CI: 1.1–17%) longer than patients without respiratory depression (*p* = 0.024). Similar results were observed upon analysis of all patients enrolled in the United States, including outliers (S6 Table), where the regression model identified a hospital length of stay 20% (95% CI: 6–35%) longer in patients with ≥1 respiratory depression episode (*p* < 0.005).

**Table 4:** Multiple regression model of hospital length of stay for patients in the United States (excluding outliers)

Clinical Characteristic	Estimate	Standard Error	Wald 95% Confidence Limits	Wald Chi-Square	Pr > Chi Square
**BMI**					
≥ 20 - < 25	0.0151	0.1060	−0.1926 - 0.2228	0.0202	.887
≥ 25 - < 30	−0.0419	0.1039	−0.2455 - 0.1617	0.1624	.687
≥ 30 - < 35	−0.0808	0.1076	−0.2917 - 0.1301	0.564	.453
≥ 35	−0.2070	0.1053	−0.4134 -0.0007	3.8658	.049
**Number of Opioids**					
> 1 - < 4	−0.2885	0.0672	−0.4202 - 0.1568	18.4243	<.0001
≥ 4	−0.3768	0.0715	−0.5171 -0.2366	27.7411	<.0001
**High risk surgery**	0.2468	0.0706	0.1084–0.3851	12.223	.0005
**Open surgery**	0.1921	0.0643	0.0661–0.3181	8.9292	.003
**Length of surgery (hr)**					
≥ 2 - < 4	0.1932	0.0468	0.1016–0.2849	17.0719	<.0001
≥ 4	0.5523	0.0491	0.4562–0.6485	126.7597	<.0001
**≥1 Respiratory Depression Episode**	0.0846	0.0374	0.0112–0.1579	5.1058	.024
**Hypertension**	−0.0964	0.0379	−0.1707 - 0.0221	6.4728	.011
**Chronic Heart Failure**	0.2964	0.1116	0.0777–0.5152	7.0546	.008
**Sepsis**	0.5316	0.1083	0.3193–0.744	24.0798	<.0001

Pearson Chi-Square/DF = 1.46

Abbreviations: *BMI* body mass index

## Discussion

This study evaluated the impact of respiratory depression on length of stay and hospital costs, which unlike the impact of general ORADEs on these outcomes, are not well described in the literature [11, 15–20]. United States patients who had ≥1 respiratory depression episode had a significantly longer length of stay and a higher cost of hospitalization, compared to patients without opioid-induced respiratory depression. Patients at high risk for respiratory depression (PRODIGY score) with ≥1 confirmed respiratory depression episode also had significantly higher hospital costs. In-depth propensity weighted analysis found that patients with ≥1 respiratory depression episode in the United States cohort had a 16% higher healthcare cost compared to patients without respiratory depression and a 17% higher healthcare cost excluding patient outliers. Total healthcare costs, which included the sum of fixed and variable costs incurred by the hospital, were significantly increased by patient length of stay, length of stay complicated by occurrence of respiratory depression, longer length of surgery, and procedure type. Importantly, respiratory depression identified by continuous capnography and pulse oximetry monitoring was critical, since patients with respiratory depression experienced exponentially increased healthcare costs as length of stay increased. In contrast, in the absence of respiratory depression episodes, increased length of stay was associated with increased healthcare cost, but this association was linear.

The hospital general care unit or floor remains the site for an alarmingly high number of acute cardiorespiratory compromise events [30]. About 290,000 in hospital cardiac arrests occur in the United States each year, of which 40% have a respiratory insufficiency etiology. These events are usually preceded by a period of 6–8 h of gradual change in vital signs, which are not detected with traditional ‘spot-check’ based monitoring as is in place today [31, 32]. A majority of opioid-induced perioperative respiratory complications therefore occur in the under-monitored hospital floor and are associated with serious patient outcomes, including anoxic brain injury and mortality, as well as legal claims with significant financial burdens [13, 33]. Universal adoption of continuous monitoring systems is an attractive option, however the initial resource expenditure, challenges of alarm fatigue, and lack of interventions based on alarm data remain at large. The PRODIGY score can help the bedside provider risk stratify patients for respiratory impairment and decide, on an individual basis, the need for continuous monitoring [4]. Other risk scores have been developed to identify patients at risk for ORADEs [19], however PRODIGY is a novel score to identify patients at risk specifically for opioid-induced respiratory depression [4].

Similar to our work, other trials have reported that ORADEs, a majority of which are respiratory, are associated with increased healthcare utilization, longer hospital length of stay, higher 30-day readmission, and increased healthcare costs [14, 15, 19–21, 33]. Studies have also demonstrated the utility of continuous pulse oximetry on the general care floor, where up to 90% of postoperative hypoxemia episodes go undetected by intermittent spot-check monitoring [5]. In one study, continuous pulse oximetry on the hospital floor reduced rescue events and ICU transfers, and hence decreased healthcare costs [34]. Similarly, after implementing continuous capnography to monitor patients receiving intravenous patient controlled analgesia opioids on the hospital floor, Stites and colleagues reported a 50% reduction in the incidence of opioid-induced respiratory depression rescue using rapid response teams, and a 79% decrease in transfers to higher levels of care, both of which are costly endeavors [35]. The PRODIGY trial confirmed a 46% incidence of opioid-induced respiratory depression using continuous pulse oximetry and capnography, which has been shown to detect respiratory depression better than pulse oximetry alone [4, 5, 8, 36]. Given the high frequency of respiratory depression and our findings that it increases healthcare utilization and cost, reducing the incidence of respiratory depression may lead to decreased length of stay and healthcare costs.

The additive cost burden of respiratory depression of $3237 (16% increase) (with outliers) and $3200 (17% increase) (without outliers) in hospitalization costs per PRODIGY trial analysis, is somewhat less than the $4350–$8225 range (27–47% increase) reported in the literature [14, 15, 17, 20]. The more conservative cost burden estimate, as found by the PRODIGY trial, may be explained by PRODIGY being a prospective trial that used continuous capnography and oximetry monitoring to identify opioid-induced respiratory depression, and required strict adherence to inclusion and exclusion criteria. In comparison, previous studies which were retrospective in nature and relied on claims analyses and coded instances of ORADEs, likely missed milder and potentially less costly cases of ORADE [14, 15, 17, 20].

Although other studies have reported differences in cost and healthcare outcomes for ORADEs, the factors contributing to these outcomes are not well described [14, 15, 19–21, 33]. Our analysis identified patient characteristics that significantly impacted length of stay and cost. Use of multiple opioids; longer, high risk, or open surgery; respiratory depression; and medical conditions including chronic heart failure, hypertension, and sepsis, all contributed to increased length of stay. Interestingly, the PRODIGY score accounts for chronic heart failure and opioid naivety when determining patient risk for respiratory depression [4]. Importantly, respiratory depression contributed to both length of stay and cost, highlighting its importance in determining patient outcomes. The findings of this trial may be of particular interest to payers (e.g., CMS), organizations related to quality measurement and reporting (e.g., National Quality Forum), and hospital administrations, highlighting the unmet need in the quality of care for general care floor patients receiving opioids, and the potential need to institute quality metrics to improve outcomes and reduce costs in this patient population. Finally, our analysis excluded outlier patients who had very high costs or an extended length of stay, and confirmed that inclusion or exclusion of these patients did not alter our main findings. Therefore, the increases in length of stay and cost for patients with respiratory depression are not due to a subset of patients requiring extended care or costly interventions but reflect differences between typical general care floor patients with and without respiratory depression.

Our work is limited by the fact that we included a portion of United States hospitals from our trial cohort, though PRODIGY also enrolled in Asia and Europe. While this may limit the generalizability of our data, we included a substantial number of United States patients and hospitals of various types and sizes. Our analysis evaluated the actual hospital cost incurred, including both fixed and variable costs, and did not rely on diagnosis related group payment data. However, this analysis was limited to the total hospital cost per patient, preventing identification of specific factors that may have contributed to increased hospital cost for patients with respiratory depression. Determination of opportunity cost and productivity loss associated with increased length of stay was out of the scope of this analysis but would be a valuable addition to future studies. Furthermore, an actual calculation of the ‘break-even’ cost of the institution of monitoring versus the cost of respiratory depression was out of the scope of this work.

## Conclusions

The improvement of surveillance monitoring on the hospital general care floor has the potential to reduce postoperative complications and lower hospital costs [34, 35, 37, 38]. Patients with opioid-induced respiratory depression episodes detected by continuous capnography and oximetry experienced a longer hospital length of stay and exponentially higher hospital costs. Future studies should explore whether early institution of these continuous monitoring measures, in combination with early proactive intervention, such as readjustment of analgesia, optimal fluid balance, aggressive incentive spirometry, and additional bronchodilation, mitigate the occurrence of respiratory depression and decrease hospital costs associated with such episodes.

## Supplementary Information

**Additional file 1: S1 Table.** Multivariable Model Prediction of Respiratory Depression, PRODIGY Scoring System, and Utilization.

**Additional file 2: S2 Table.** Demographic and clinical characteristics before and after propensity weighting of all enrolled patients in the United States.

**Additional file 3: S3 Table.** Demographic and clinical characteristics before and after propensity weighting of enrolled patients in the United States, excluding outliers.

**Additional file 4: S4 Table.** Surgical procedures performed on enrolled patients in the United States.

**Additional file 5: S5 Table.** Generalized linear model of healthcare costs in all enrolled United States patients, including patient outliers.

**Additional file 6: S6 Table.** Multiple regression model of hospital length of stay for all enrolled patients in the United States, including patient outliers.

## Data Availability

The datasets supporting the conclusions of this article are included within the article (and its additional files).

## Abbreviations

ASA: American Society of Anesthesiologists
BMI: Body Mass Index
CCR: cost to charge ratio
CI: Confidence Interval
CMS: Centers for Medicare and Medicaid
CNS: Central Nervous System
ICU: Intensive Care Unit
ORADE: Opioid Related Adverse Drug Event
PRODIGY: PRediction of Opioid-induced respiratory Depression In patients monitored by capnoGraphY
SD: Standard Deviation
USD: United States Dollars
