# Supplementary material for: Opioid-induced respiratory depression increases hospital costs and length of stay in patients recovering on the general care floor
Source: BMC Anesthesiol. 2021 Mar 20;21:88. doi: 10.1186/s12871-021-01307-8 (PMC7980593; doi:10.1186/s12871-021-01307-8)
Supplement: Supplementary file 1 — Additional file 1: S1 Table. Multivariable Model Prediction of Respiratory Depression, PRODIGY Scoring System, and Utilization. [file 12871_2021_1307_MOESM1_ESM.pdf]

S1 Table. Multivariable Model Prediction of Respiratory Depression, PRODIGY Scoring System, and Utilization. Adapted with permission from Wolters Kluwer Health, Inc: Khanna AK, Bergese SD, Jungquist CR, et al. Prediction of Opioid-Induced Respiratory Depression on Inpatient Wards Using Continuous Capnography and Oximetry: An International Prospective, Observational Trial. *Anesth Analg*; Publish Ahead of Print April 16, 2020. doi: 10.1213/ANE.0000000000004788. URL: https://journals.lww.com/anesthesia-analgesia/Abstract/9000/Prediction_of_Opioid_Induced_Respiratory.95670.aspx

	Multivariable Model Predictors				Points if Clinical Characteristic = "Yes"
**Clinical Characteristic**	**Estimate**	**Standard Error**	**OR (95% CI)**	**Pr > |t|**	
Age (y)					
<60	Reference	...	...	...	0
≥60 to <70	0.8077	0.1458	2.243 (1.685–2.985)	<0.0001	8
≥70 to <80	1.2323	0.1805	3.429 (2.407–4.886)	<0.0001	12
≥80	1.5647	0.3657	4.781 (2.333–9.798)	<0.0001	16
Sex (M)	0.7550	0.1284	2.128 (1.654–2.737)	<0.0001	8
Opioid naive	0.2912	0.1652	1.338 (0.968–1.850)	0.0782	3
Sleep disorders	0.4755	0.1998	1.609 (1.087–2.381)	0.0175	5
Chronic heart failure	0.7494	0.4085	2.116 (0.949–4.715)	0.0668	7
					"Sum = PRODIGY Score"

	PRODIGY Score Distribution			
	**Low Risk**	**Intermediate Risk**	**High Risk**	***P***
PRODIGY score	<8 points	≥8 and <15 points	≥15 points	
% Pts with respiratory depression in risk category (n Pts in risk category with respiratory depression/n Pts in risk category)	24% (83/351)	42% (192/457)	65% (299/458)	<.0001
Sensitivity (95% CI)	...	0.86 (0.82–0.88)	0.52 (0.48–0.56)	
Specificity (95% CI)	...	0.39 (0.35–0.42)	0.77 (0.74–0.80)	
PPV (95% CI)	...	0.54 (0.50–0.57)	0.65 (0.61–0.70)	
NPV (95% CI)	...	0.76 (0.72–0.81)	0.66 (0.63–0.69)	
OR (95% CI; *P*)	OR_{IL} =2.34 (1.72–3.19; *P* < .001) OR_{HL} = 6.07 (4.44–8.30; *P* < .001)	OR_{HI} =2.6 (1.99–3.39; *P* < .001)		

Abbreviations: CI, confidence interval; M, male; NPV, negative predictive value; OR, odds ratio; OR_{HI}, odds ratio, high- versus intermediate-risk groups; OR_{HL}, odds ratio, high- versus low-risk groups; OR_{IL}, odds ratio, intermediate- versus low-risk groups; PPV, positive predictive value; PRODIGY, PRediction of Opioid-induced respiratory Depression In patients monitored by capnoGraphY; Pts, patients.
