# Supplementary material for: Opioid-induced respiratory depression increases hospital costs and length of stay in patients recovering on the general care floor
Source: BMC Anesthesiol. 2021 Mar 20;21:88. doi: 10.1186/s12871-021-01307-8 (PMC7980593; doi:10.1186/s12871-021-01307-8)
Supplement: Supplementary file 2 — Additional file 2: S2 Table. Demographic and clinical characteristics before and after propensity weighting of all enrolled patients in the United States. [file 12871_2021_1307_MOESM2_ESM.pdf]

S2 Table. Demographic and clinical characteristics before and after propensity weighting of all enrolled patients in the United States.

	Patient Characteristics before Propensity Weighting			Patient Characteristics after Propensity Weighting		
**Clinical Characteristic**	**No Respiratory Depression Episode (n=272)**	**≥1 Respiratory Depression Episode (n=148)**	**p-value**	**No Respiratory Depression Episode (n=255)[a]**	**≥1 Respiratory Depression Episode (n=128)[a]**	**p-value**
**Age (yr)** (Mean ± SD)	52.4 ± 14.1	62.4 ± 11.5		55.4 ± 14.1	58.1 ± 11.2	
<60	67.6% (184/272)	38.5% (57/148)	<.0001	57.7% (147/255)	56.9% (73/128)	.996
≥60 - <70	20.6% (56/272)	34.5% (51/148)		25.8% (66/255)	25.8% (33/128)	
≥70 - <80	9.9% (27/272)	20.9% (31/148)		13.3% (34/255)	13.7% (18/128)	
≥80	1.8% (5/272)	6.1% (9/148)		3.2% (8/255)	3.6% (5/128)	
**Sex (Male)**	30.9% (84/272)	50.7% (75/148)	<.0001	36.7% (94/255)	44.5% (57/128)	.141
**BMI (kg/m^2)** (Mean ± SD)	32.4 ± 9.6	30.2 ± 6.1				
<20	3.7% (10/272)	2% (3/148)	.023	1.9% (5/255)	1.1% (1/128)	.748
≥20 - <25	16.9% (46/272)	16.9% (25/148)		18% (46/255)	19.8% (25/128)	
≥25 - <30	27.6% (75/272)	35.1% (52/148)		30.4% (77/255)	27.8% (36/128)	
≥30 - <35	19.5% (53/272)	27% (40/148)		21.6% (55/255)	26.6% (34/128)	
≥35	32.4% (88/272)	18.9% (28/148)		28.2% (72/255)	24.8% (32/128)	
**Race/Ethnicity**						
American Indian or Alaska Native	0.4% (1/272)	0% (0/148)	<.0001	0.3% (1/255)	0% (0/128)	.904
Asian	1.1% (3/272)	0% (0/148)		0.8% (2/255)	0% (0/128)	
Black or African American	30.9% (84/272)	12.8% (19/148)		24% (61/255)	28.1% (36/128)	
Hispanic	0.7% (2/272)	0.7% (1/148)		0.5% (1/255)	0% (0/128)	
White	66.2% (180/272)	85.8% (127/148)		73.7% (188/255)	71.2% (91/128)	
Other	0.7% (2/272)	0.7% (1/148)		0.8% (2/255)	0.7% (1/128)	
**Current Smoker**	16.5% (45/272)	14.9% (22/148)	.679	15.5% (40/255)	18.2% (23/128)	.505
**Neck circumference**	47.6% (129/271)	41.5% (61/147)	.258	46.5% (119/255)	43.9% (56/128)	.633
**ASA Physical Status**						
ASA I	0.4% (1/262)	0.7% (1/145)	.390	0.7% (2/255)	0.7% (1/128)	.750
ASA II	42.4% (111/262)	35.9% (52/145)		40.6% (103/255)	42.8% (55/128)	
ASA III	55% (144/262)	59.3% (86/145)		56.4% (144/255)	54.1% (69/128)	
ASA IV	2.3% (6/262)	4.1% (6/145)		2.3% (6/255)	2.3% (3/128)	
**Surgery Demographics**						
Surgical Patient	94.1% (256/272)	95.9% (142/148)	.498	97.8% (249/255)	97% (124/128)	.737
High Risk Surgery	4.8% (13/272)	7.4% (11/148)	.277	4.4% (11/255)	4.6% (6/128)	.948
Open Surgery	7.7% (21/272)	12.8% (19/148)	.116	10.3% (26/255)	10% (13/128)	.938
**Length of Surgery (hr)**						
0	5.9% (16/272)	4.1% (6/148)		2.2% (6/255)	3% (4/128)	
<2	36.4% (99/272)	27% (40/148)	.010	34.9% (89/255)	33.4% (43/128)	.961
≥2 - <4	44.1% (120/272)	48.6% (72/148)		46.6% (119/255)	47.7% (61/128)	

	Patient Characteristics before Propensity Weighting			Patient Characteristics after Propensity Weighting		
**Clinical Characteristic**	**No Respiratory Depression Episode (n=272)**	**≥1 Respiratory Depression Episode (n=148)**	**p-value**	**No Respiratory Depression Episode (n=255)[a]**	**≥1 Respiratory Depression Episode (n=128)[a]**	**p-value**
≥4	13.6% (37/272)	20.3% (30/148)		16.3% (42/255)	16% (20/128)	
**Opioid Demographics**						
Opioid Naive	68.8% (187/272)	79.1% (117/148)	.023	73% (186/255)	77.4% (99/128)	.361
Multiple Opioids or concurrent CNS/Sedating Medication	98.9% (269/272)	98% (145/148)	.430	100% (255/255)	100% (128/128)	N/A
One opioid	2.6% (7/272)	2.7% (4/148)	.358	0.9% (2/255)	0.7% (1/128)	.753
Opioid number >1 - <4	49.6% (135/272)	56.8% (84/148)		51.9% (132/255)	56.2% (72/128)	
Opioid number ≥4	47.8% (130/272)	40.5% (60/148)		47.2% (120/255)	43.1% (55/128)	
**Cardiac Disorders**						
Aortic Aneurysm	0.4% (1/272)	3.4% (5/148)	.022	0.3% (1/255)	1.4% (2/128)	.260
Aortic Valve Disease	0.4% (1/272)	0% (0/148)	1	0.3% (1/255)	0% (0/128)	1
Chronic Heart Failure	0.7% (2/272)	2% (3/147)	.349	0.9% (2/255)	1.2% (1/128)	1
Coronary Artery Disease	2.2% (6/272)	9.6% (14/146)	.001	6.1% (16/255)	5.4% (7/128)	.781
Hypertension	48.5% (132/272)	54.7% (81/148)	.261	51.1% (130/255)	51.7% (66/128)	.909
Mitral Valve Disease	1.8% (5/272)	0.7% (1/148)	.670	1.4% (4/255)	0.5% (1/128)	.668
Myocardial Infarction	0.7% (2/272)	4.1% (6/147)	.025	2.6% (7/255)	2.3% (3/128)	1
Pulmonary Hypertension	0.7% (2/272)	0.7% (1/148)	1	0% (0/255)	0% (0/128)	---
**Sarcoidosis**	0.4% (1/272)	0% (0/148)	1	0.4% (1/266)	0% (0/137)	1
**Sepsis**	0.7% (2/272)	0.7% (1/148)	1	0.3% (1/255)	0% (0/128)	1
**Diabetes - Type I**	1.5% (4/272)	3.4% (5/148)	.289	2.6% (7/255)	1.9% (2/128)	.724
**Diabetes - Type II**	14% (38/272)	18.2% (27/148)	.261	15.8% (40/255)	18.3% (24/128)	.534
**Muscular Dystrophy**	0.4% (1/272)	0.7% (1/148)	1	0.4% (1/255)	0.5% (1/128)	1
**Kidney Failure**	1.5% (4/272)	1.4% (2/148)	1	1.2% (3/255)	0.8% (1/128)	1
**Respiratory, Thoracic and Mediastinal Disorders**						
Acute Bronchitis	2.6% (7/272)	1.4% (2/148)	.503	2.2% (6/255)	2.3% (3/128)	1
Asthma	16.9% (46/272)	10.8% (16/148)	.113	13.7% (35/255)	16.1% (21/128)	.996
Chronic Bronchitis	0.7% (2/272)	0% (0/148)	.543	0.5% (1/255)	0% (0/128)	1
Chronic Obstructive Pulmonary	4.4% (12/272)	6.8% (10/148)	.360	5.8% (15/255)	4.4% (6/128)	.556
Chronic Restrictive Lung Disease	0% (0/272)	0.7% (1/148)	.352	0% (0/255)	0% (0/128)	---
Pneumonia	0.7% (2/272)	1.4% (2/148)	.616	0.8% (2/255)	0.9% (1/128)	1
Pulmonary Fibrosis	0.4% (1/272)	0% (0/148)	1	0.3% (1/255)	0% (0/128)	1
**Sleep Disorders**	14.6% (39/268)	15.3% (22/144)	.885	14.3% (36/255)	12.9% (16/128)	.708
**Vascular Disorders**						
Cerebral Aneurysm	0.7% (2/272)	2% (3/148)	.350	1.3% (3/255)	1.1% (1/128)	.668
Peripheral Vascular Disease	1.8% (5/272)	2% (3/148)	1	1.6% (4/255)	1.2% (2/128)	1
Stroke	1.5% (4/272)	2.7% (4/147)	.459	1.7% (4/255)	1.6% (2/128)	.265
Transient Ischemic Attack	0.7% (2/272)	2% (3/147)	.349	1% (3/255)	1.2% (2/128)	1
**STOP BANG Score**						

	Patient Characteristics before Propensity Weighting			Patient Characteristics after Propensity Weighting		
**Clinical Characteristic**	**No Respiratory Depression Episode (n=272)**	**≥1 Respiratory Depression Episode (n=148)**	**p-value**	**No Respiratory Depression Episode (n=255)[a]**	**≥1 Respiratory Depression Episode (n=128)[a]**	**p-value**
Low Risk (0-2)	48.9% (130/266)	37.1% (53/143)	.033	42.6% (109/255)	41.6% (53/128)	.968
Intermediate Risk (3-4)	33.1% (88/266)	35.7% (51/143)		37.1% (95/255)	37.2% (48/128)	
High Risk (5-8)	18% (48/266)	27.3% (39/143)		20.3% (52/255)	21.2% (27/128)	
**Procedure**						
Bone and joint	22.1% (60/272)	18.2% (27/148)	.158	20.4% (52/255)	18.8% (24/128)	.978
Gastrointestinal	24.3% (66/272)	16.2% (24/148)		21.8% (56/255)	18.3% (23/128)	
Hepatobiliary	2.2% (6/272)	7.4% (11/148)		2.8% (7/255)	4.1% (5/128)	
Medical	5.9% (16/272)	4.1% (6/148)		2.2% (6/255)	3% (4/128)	
Nervous system, skull and spine	36% (98/272)	43.2% (64/148)		42.3% (108/255)	44.1% (57/128)	
Obstetric and gynecological	5.5% (15/272)	6.8% (10/148)		6.8% (17/255)	6.2% (8/128)	
Other	1.1% (3/272)	2.7% (4/148)		1% (3/255)	1.4% (2/128)	
Renal and urinary tract	0.4% (1/272)	0% (0/148)		0.3% (1/255)	0 (0/128)	
Respiratory tract	0.4% (1/272)	0% (0/148)		0.3% (1/255)	0% (0/128)	
Therapeutic procedures and supportive care	2.2% (6/272)	1.4% (2/148)		2.1% (5/255)	4.1% (5/128)	

[a]Patients missing any demographic or clinical data used excluded from propensity weighting

Abbreviations: ASA = American Society of Anesthesiologists; BMI = body mass index; CNS = central nervous system; SD= standard deviation; STOP BANG = Snoring, Tiredness, Observed apnea, blood Pressure, Body mass index, Age, Neck circumference and Gender
