# Supplementary material for: Opioid-induced respiratory depression increases hospital costs and length of stay in patients recovering on the general care floor
Source: BMC Anesthesiol. 2021 Mar 20;21:88. doi: 10.1186/s12871-021-01307-8 (PMC7980593; doi:10.1186/s12871-021-01307-8)
Supplement: Supplementary file 3 — Additional file 3: S3 Table. Demographic and clinical characteristics before and after propensity weighting of enrolled patients in the United States, excluding outliers. [file 12871_2021_1307_MOESM3_ESM.pdf]

S3 Table. Demographic and clinical characteristics before and after propensity weighting of enrolled patients in the United States, excluding outliers.

	Patient Characteristics before Propensity Weighting (excluding outliers)			Patient Characteristics after Propensity Weighting (excluding outliers)		
**Clinical Characteristic**	**No Respiratory Depression Episode (n=266)**	**≥1 Respiratory Depression Episode (n=143)**	**p-value**	**No Respiratory Depression Episode (n=247)[a]**	**≥1 Respiratory Depression Episode (n=123)[a]**	**p-value**
**Age (yr)** (Mean ± SD)	52.6 ± 14.0	62 ± 11.2		55.4 ± 13.8	58.2 ± 11	
<60	67.3% (179/266)	39.9% (57/143)	<.0001	58.4% (144/247)	56.4% (69/123)	.980
≥60 - <70	21.1% (56/266)	33.6% (48/143)		25.3% (63/247)	26.2% (32/123)	
≥70 - <80	9.8% (26/266)	21.7% (31/143)		13.6% (34/247)	14% (17/123)	
≥80	1.9% (5/266)	4.9% (7/143)		2.7% (7/247)	3.3% (4/123)	
**Sex (Male)**	30.5% (81/266)	50.3% (72/143)	.0001	36.3% (90/247)	43.3% (53/123)	.193
**BMI (kg/m^2)** (Mean ± SD)	32.4 ± 9.6	30.2 ± 6.1				
<20	3.4% (9/266)	2.1% (3/143)	.012	1.6% (4/247)	1% (1/123)	.806
≥20 - <25	17.3% (46/266)	16.8% (24/143)		18% (44/247)	19.4% (24/123)	
≥25 - <30	26.7% (71/266)	35.7% (51/143)		30.1% (74/247)	27.5% (34/123)	
≥30 - <35	19.5% (52/266)	27.3% (39/143)		21.7% (54/247)	26.5% (33/123)	
≥35	33.1% (88/266)	18.2% (26/143)		28.7% (71/247)	25.6% (32/123)	
**Race/Ethnicity**						
American Indian or Alaska Native	0.4% (1/266)	0% (0/143)	.0002	0.3% (1/247)	0% (0/123)	.931
Asian	1.1% (3/266)	0% (0/143)		0.8% (2/247)	0% (0/123)	
Black or African American	30.1% (80/266)	12.6% (18/143)		23.9% (59/247)	27.1% (33/123)	
Hispanic	0.8% (2/266)	0.7% (1/143)		0.5% (1/247)	0% (0/123)	
White	0.8% (2/266)	0.7% (1/143)		0.8% (2/247)	0.7% (1/123)	
Other	66.9% (178/266)	86% (123/143)		73.7% (182/247)	72.2% (89/123)	
**Current Smoker**	16.5% (44/266)	15.4% (22/143)	.762	15.9% (39/247)	17.6% (22/123)	.672
**Neck circumference**	47.9% (127/265)	40.8% (58/142)	.258	46.6% (115/247)	44.5% (55/123)	.708
**ASA Physical Status**						
ASA I	0.4% (1/256)	0.7% (1/140)	.364	0.7% (2/247)	0.8% (1/123)	.952
ASA II	42.2% (108/256)	36.4% (51/140)		40.5% (100/247)	43% (53/123)	
ASA III	55.9% (143/256)	59.3% (83/140)		56.9% (141/247)	54.2% (67/123)	
ASA IV	1.6% (4/256)	3.6% (5/140)		1.9% (5/247)	2% (2/123)	
**Surgery Demographics**						
Surgical Patient	94.4% (251/266)	96.5% (138/143)	.338	98% (242/247)	96.9% (119/123)	.478
High Risk Surgery	4.9% (13/266)	7.7% (11/143)	.250	4.5% (11/247)	4.7% (6/123)	.951
Open Surgery	7.5% (20/266)	13.3% (19/143)	.058	10.3% (26/247)	10.1% (12/123)	.934
**Length of Surgery (hr)**						
0 hour	5.9% (16/272)	4.1% (6/148)		2% (5/247)	3.1% (4/123)	
<2	36.4% (99/272)	27% (40/148)	.085	34.7% (86/247)	33.6% (41/123)	.914
≥2 - <4	44.1% (120/272)	48.6% (72/148)		46.8% (116/247)	46.8% (58/123)	

Clinical Characteristic	Patient Characteristics before Propensity Weighting (excluding outliers)			Patient Characteristics after Propensity Weighting (excluding outliers)		
	No Respiratory Depression Episode (n=266)	≥1 Respiratory Depression Episode (n=143)	p-value	No Respiratory Depression Episode (n=247)[a]	≥1 Respiratory Depression Episode (n=123)[a]	p-value
≥4	13.6% (37/272)	20.3% (30/148)		16.5% (41/247)	16.5% (20/123)	
**Opioid Demographics**						
Opioid Naive	68.8% (183/266)	79.7% (114/143)	.018	73.4% (182/247)	77.5% (95/123)	.401
Multiple Opioids or concurrent CNS/Sedating Medication	98.9% (263/266)	97.9% (140/143)	.426	100% (247/247)	99.7% (123/123)	1
One opioid	2.6% (7/266)	2.8% (4/143)	.334	0.9% (2/247)	0.7% (1/123)	.780
Opioid number >1 - <4	49.2% (131/266)	56.6% (81/143)		52.2% (129/247)	56.5% (70/123)	
Opioid number ≥4	48.1% (128/266)	40.6% (58/143)		46.9% (116/247)	42.8% (53/123)	
**Cardiac Disorders**						
Aortic Aneurysm	0.4% (1/266)	3.5% (5/143)	.021	0.3% (1/247)	1.4% (2/123)	1
Aortic Valve Disease	0.4% (1/266)	0% (0/148)	1	0% (0/247)	0% (0/123)	---
Chronic Heart Failure	0.4% (1/266)	2.1% (3/142)	.124	0.6% (2/247)	1.1% (1/123)	1
Coronary Artery Disease	2.3% (6/266)	8.5% (12/141)	.004	5.1% (13/247)	5.1% (6/123)	.997
Hypertension	48.9% (130/266)	53.8% (77/143)	.337	50.7% (125/247)	51.3% (63/123)	.908
Mitral Valve Disease	1.5% (4/266)	0.7% (1/143)	.662	1.2% (3/247)	0.4% (1/123)	1
Myocardial Infarction	0.8% (2/266)	3.5% (5/142)	.052	2.2% (5/247)	2.1% (3/123)	1
Orthostatic Hypotension	0% (0/272)	0% (0/148)	---	0% (0/247)	0% (0/123)	---
Pulmonary Hypertension	0.8% (2/266)	0% (0/148)	.544	0% (0/247)	0% (0/123)	---
**Sarcoidosis**	0.4% (1/266)	0% (0/148)	1	0% (0/247)	0% (0/123)	---
**Sepsis**	0.8% (2/266)	0.7% (1/143)	1	0.4% (1/247)	0.4% (1/123)	1
**Diabetes - Type I**	1.5% (4/266)	3.5% (5/143)	.287	2.2% (6/247)	2% (2/123)	1
**Diabetes - Type II**	13.9% (37/266)	17.5% (25/143)	.337	15.7% (39/247)	18.7% (23/123)	.470
**Muscular Dystrophy**	0.4% (1/266)	0.7% (1/143)	1	0.4% (1/247)	0.4% (1/123)	1
**Kidney Failure**	1.1% (3/266)	1.4% (2/143)	1	1% (2/247)	0.8% (1/123)	1
**Respiratory, Thoracic and Mediastinal Disorders**						
Acute Bronchitis	2.6% (7/266)	1.4% (2/143)	.504	2.3% (6/247)	2.5% (3/123)	1
Asthma	16.2% (43/266)	11.2% (16/143)	.187	13.4% (33/247)	14.8% (18/123)	.707
Chronic Bronchitis	0.8% (2/266)	0% (0/148)	.544	0.5% (1/247)	0% (0/123)	1
Chronic Obstructive Pulmonary	4.5% (12/266)	6.3% (9/143)	.436	5.5% (14/247)	4.5% (6/123)	.679
Chronic Restrictive Lung Disease	0% (0/272)	0.7% (1/143)	.350	0% (0/247)	0% (0/123)	---
Pneumonia	0.8% (2/266)	1.4% (2/143)	.614	0.8% (2/247)	0.9% (1/123)	1
Pulmonary Fibrosis	0.4% (1/266)	0% (0/148)	1	0% (0/247)	0% (0/123)	---
**Sleep Disorders**	14.5% (38/262)	15.8% (22/139)	.724	14.5% (36/247)	13.4% (16/123)	.776
**Vascular Disorders**						
Cerebral Aneurysm	0.8% (2/266)	2.1% (3/143)	.348	1.3% (3/247)	1.1% (1/123)	1
Peripheral Vascular Disease	1.9% (5/266)	2.1% (3/143)	1	1.6% (4/247)	1.4% (2/123)	1
Stroke	1.1% (3/266)	2.8% (4/142)	.244	1.6% (4/247)	1.6% (2/123)	1

	Patient Characteristics before Propensity Weighting (excluding outliers)			Patient Characteristics after Propensity Weighting (excluding outliers)		
**Clinical Characteristic**	**No Respiratory Depression Episode (n=266)**	**≥1 Respiratory Depression Episode (n=143)**	**p-value**	**No Respiratory Depression Episode (n=247)[a]**	**≥1 Respiratory Depression Episode (n=123)[a]**	**p-value**
Transient Ischemic Attack	0.8% (2/266)	1.4% (2/142)	.613	0.9% (2/247)	1% (1/123)	1
**STOP BANG Score**						
Low Risk (0-2)	48.5% (126/260)	37% (51/138)	.0374	43% (106/247)	40.6% (50/123)	.904
Intermediate Risk (3-4)	33.5% (87/260)	35.5% (49/138)		36.6% (90/247)	37.6% (46/123)	
High Risk (5-8)	18.1% (47/260)	27.5% (38/138)		20.4% (50/247)	21.8% (27/123)	
**Procedure**			.154			.985
Bone and joint	21.8% (58/266)	18.2% (26/143)		20.8% (51/247)	18.8% (23/123)	
Gastrointestinal	24.8% (66/266)	16.8% (24/143)		22.5% (56/247)	19.1% (24/123)	
Hepatobiliary	2.3% (6/266)	7.7% (11/143)		3% (7/247)	4.3% (5/123)	
Medical	5.6% (15/266)	3.5% (5/143)		2% (5/247)	3.1% (4/123)	
Nervous system, skull and spine	35.7% (95/266)	43.4% (62/143)		41.2% (102/247)	43.1% (53/123)	
Obstetric and gynecological	5.6% (15/266)	6.3% (9/143)		6.8% (17/247)	6.4% (8/123)	
Other	1.1% (3/266)	2.8% (4/143)		1.1% (3/247)	1.5% (2/123)	
Renal and urinary tract	0.4% (1/266)	0% (0/143)		0.3% (1/247)	0% (0/123)	
Respiratory tract	0.4% (1/266)	0% (0/143)		0.3% (1/247)	0% (0/123)	
Therapeutic procedures and supportive care	2.3% (6/266)	1.4% (2/143)		2.2% (5/247)	3.7% (5/123)	

[a]Patients missing any demographic or clinical data used excluded from propensity weighting

Abbreviations: ASA = American Society of Anesthesiologists; BMI = body mass index; CNS = central nervous system; SD= standard deviation; STOP BANG = Snoring, Tiredness, Observed apnea, blood Pressure, Body mass index, Age, Neck circumference and Gender
