# Supplementary material for: Opioid-induced respiratory depression increases hospital costs and length of stay in patients recovering on the general care floor
Source: BMC Anesthesiol. 2021 Mar 20;21:88. doi: 10.1186/s12871-021-01307-8 (PMC7980593; doi:10.1186/s12871-021-01307-8)
Supplement: Supplementary file 4 — Additional file 4: S4 Table. Surgical procedures performed on enrolled patients in the United States. [file 12871_2021_1307_MOESM4_ESM.pdf]

**S4 Table. Surgical procedures performed on enrolled patients in the United States.**

| Procedure Type | Percent of Procedures |
|---|---|
| Bone and joint | 20.71% |
| Gastrointestinal | 21.43% |
| Hepatobiliary | 4.05% |
| Medical | 5.24% |
| Nervous system, skull and spine | 38.57% |
| Obstetric and gynecological | 5.95% |
| Renal and urinary tract | 0.24% |
| Respiratory tract | 0.24% |
| Therapeutic procedures and supportive care | 1.9% |
| Other | 1.67% |
