# Supplementary material for: Opioid-induced respiratory depression increases hospital costs and length of stay in patients recovering on the general care floor
Source: BMC Anesthesiol. 2021 Mar 20;21:88. doi: 10.1186/s12871-021-01307-8 (PMC7980593; doi:10.1186/s12871-021-01307-8)
Supplement: Supplementary file 5 — Additional file 5: S5 Table. Generalized linear model of healthcare costs in all enrolled United States patients, including patient outliers. [file 12871_2021_1307_MOESM5_ESM.pdf]

S5 Table. Generalized linear model of healthcare costs in all enrolled United States patients, including patient outliers.

Clinical Characteristic	Exponentiated estimates	95% CI	p value
**Intercept**	8,002.43	3,589.61 – 17,840.03	<.0001
**Length of stay**	1.06	1.05 – 1.07	<.0001
**Respiratory depression**	0.92	0.80 – 1.06	.271
**Length of stay* Respiratory depression**	1.04	1.01 – 1.06	.002
**Open Surgery (vs laparoscopic)**	0.92	0.79 – 1.08	.309
**Length of surgery (hr)**			<.0001
0	0.75	0.53 – 1.05	
≥2 - <4 vs. <2	1.28	1.17 – 1.41	
>4 vs. <2	1.71	1.51 – 1.93	
**BMI**			.002
<20	0.82	0.63 - 1.06	
≥20 - <25	---	---	---
≥25 - <30	0.94	0.83 - 1.05	
≥30 - <35	1	0.88 - 1.13	
≥35	1.19	1.03 - 1.38	
**Procedure**			<.0001
Bone and joint	1.42	1.06 – 1.92	
Gastrointestinal	1.03	0.77 – 1.38	
Hepatobiliary	1.31	0.91 – 1.89	
Nervous system, skull and spine	1.81	1.35 – 2.41	
Obstetric and gynecological	1.10	0.80 – 1.51	
Renal and urinary tract	2.07	0.93 – 4.58	
Respiratory tract	1.10	0.48 – 2.52	
Other	1.56	0.95 – 2.57	
Therapeutic procedures and supportive care	---	---	---
Medical[a]	0.61	0.42 – 0.88	.008

[a]Effect of medical procedure was estimated in a separate model due to the multicollinearity between length of surgery and medical patients.

Abbreviations: 95% CI = 95% confidence interval; BMI = body mass index
