# Supplementary material for: Opioid-induced respiratory depression increases hospital costs and length of stay in patients recovering on the general care floor
Source: BMC Anesthesiol. 2021 Mar 20;21:88. doi: 10.1186/s12871-021-01307-8 (PMC7980593; doi:10.1186/s12871-021-01307-8)
Supplement: Supplementary file 6 — Additional file 6: S6 Table. Multiple regression model of hospital length of stay for all enrolled patients in the United States, including patient outliers. [file 12871_2021_1307_MOESM6_ESM.pdf]

S6 Table. Multivariable regression model of hospital length of stay for all enrolled patients in the United States, including patient outliers.

Clinical Characteristic	Estimate	Standard Error	Wald 95% Confidence Limits	Wald Chi-Square	Pr > Chi Square
**Male**	0.1759	0.0621	0.0542 - 0.2977	8.03	.005
**BMI**					
≥20 - <25	-0.2536	0.1613	-0.5698 - 0.0626	2.47	.116
≥25 - <30	-0.0974	0.1565	-0.4042 - 0.2094	0.39	.534
≥30 - <35	-0.1261	0.1636	-0.4467 - 0.1944	0.59	.441
≥35	-0.2105	0.1605	-0.5251 - 0.1041	1.72	.190
**Opioid Naive**	-0.2907	0.0681	-0.4242 - -0.1572	18.21	<.0001
**Number of Opioids**					
>1 - <4	-0.5788	0.0884	-0.7522 - -0.4055	42.85	<.0001
≥4	-0.8847	0.1043	-1.0892 - -0.6802	71.93	<.0001
**Length of surgery (hr)**					
≥2 - <4	-0.1368	0.0703	-0.2746 - 0.0009	3.79	.052
≥4	0.1668	0.0740	0.0217 - 0.3119	5.08	.024
**Current smoker**	0.2363	0.0782	0.0830 - 0.3895	9.13	.003
**Sleep disorders**	-0.1042	0.0856	-0.2719 - 0.0635	1.48	.223
**≥1 Respiratory Depression Episode**	0.1779	0.0607	0.0590 - 0.2968	8.60	.003
**Aortic valve disease**	-0.3277	0.2373	-0.7927 - 0.1373	1.91	.167
**Hypertension**	-0.0808	0.0611	-0.2005 - 0.0389	1.75	.186
**Myocardial infarction**	0.2484	0.1541	-0.0537 - 0.5505	2.60	.107
**Acute bronchitis**	0.3494	0.1833	-0.0099 - 0.7087	3.63	.057
**Asthma**	-0.0937	0.0909	-0.2719 - 0.0845	1.06	.303
**Peripheral Vascular Disease**	0.3927	0.1532	0.0923 - 0.6930	6.57	.010
**Transient ischemic attack**	-0.3266	0.2335	-0.7843 - 0.1310	1.96	.162
**Kidney Failure**	0.1708	0.1311	-0.0861 - 0.4278	1.70	.193
**Sepsis**	1.2003	0.1263	0.9527 - 1.4479	90.27	<.0001

Abbreviation: BMI = body mass index
